# Depression and anxiety in epilepsy: the association with demographic and seizure-related variables

**DOI:** 10.1186/1744-859X-6-28

**Published:** 2007-10-30

**Authors:** Vasilios K Kimiskidis, Nikolaos I Triantafyllou, Eleni Kararizou, Stergios-Stylianos Gatzonis, Konstantinos N Fountoulakis, Anna Siatouni, Panagiotis Loucaidis, Dimitra Pseftogianni, Nikolaos Vlaikidis, George S Kaprinis

**Affiliations:** 1 Aristotle University of Thessaloniki, Department of Neurology III, Thessaloniki, Greece; 2 University of Athens, Neurological Clinic, Eginition Hospital, Athens, Greece; 3 University of Athens, Department of Neurosurgery, Athens, Greece; 4 Aristotle University of Thessaloniki, Department of Psychiatry III, Thessaloniki, Greece

## Abstract

**Background:**

Depression and anxiety are common psychiatric symptoms in patients with epilepsy, exerting a profound negative effect on health-related quality of life. Several issues, however, pertaining to their association with psychosocial, seizure-related and medication factors, remain controversial. Accordingly, the present study was designed to investigate the association of interictal mood disorders with various demographic and seizure-related variables in patients with newly-diagnosed and chronic epilepsy.

**Methods:**

We investigated 201 patients with epilepsy (51.2% males, mean age 33.2 ± 10.0 years, range 16–60) with a mean disease duration of 13.9 ± 9.5 years. Depression and anxiety were assessed in the interictal state with the Beck Depression Inventory, 21-item version (BDI-21) and the state and trait subscales of the State-Trait Anxiety Inventory (STAI-S and STAI-T), respectively. The association of mood disorders with various variables was investigated with simple and multiple linear regression analyses.

**Results:**

High seizure frequency and symptomatic focal epilepsy (SFE) were independent determinants of depression, together accounting for 12.4% of the variation of the BDI-21. The STAI-S index was significantly associated with the type of epilepsy syndrome (SFE). Finally, high seizure frequency, SFE and female gender were independent determinants of trait anxiety accounting for 14.7% of the variation of the STAI-T.

**Conclusion:**

Our results confirm the prevailing view that depression and anxiety are common psychological disorders in epileptics. It is additionally concluded that female gender, high seizure frequency and a symptomatic epilepsy syndrome are independent risk factors for the development of anxiety and/or depression.

## Background

Despite the fact that some patients with epilepsy lead normal lives, devoid of cognitive or emotional problems, a significant number of them experience psychiatric disturbances, including mood disorders. Amongst the latter, depression is the most extensively studied with a large number of controlled studies reporting prevalence rates ranging from 3–55% [1]. Anxiety might be even more common, occurring in 25% of epileptic subjects in a community setting (versus 9% classified as depressed) [2] whereas in secondary care and specialist centers its prevalence exceeds 50% [3,4]. Of particular clinical importance is the recent finding that depression and anxiety exert a profound negative effect on the health-related quality of life (HRQOL) in patients with epilepsy. For instance, in a study by Choi-Kwon et al [5], depression and anxiety explained more variance in HRQOL than did any other seizure-related or demographic variable.

While the impact of mood disorders on HRQOL in epilepsy is now well established, several other issues pertaining to their association with psychosocial, seizure-related and medication factors, remain controversial. For instance, gender [6-8] and seizure etiology [9-12] have been variously reported as being significantly or non-significantly associated with mood disorders, most likely due to methodological differences amongst relevant studies. The resolution of these controversies is not only of theoretical but also of practical importance, as a clear understanding of the complex pathogenesis of mood disorders in epilepsy is a prerequisite for the development of effective intervention strategies.

Accordingly, the present study was designed to investigate the association of interictal mood disorders with various demographic and seizure-related variables in patients with newly-diagnosed and chronic epilepsy.

## Methods

Patients attending the Outpatient Epilepsy Clinics of three University Hospitals entered this study after giving informed consent for the procedures that were approved by an institutional review board.

All participants were previously subjected to a thorough clinical and laboratory investigation, including electroencephalogram (EEG) and high-resolution brain magnetic resonance imaging (MRI) scanning, so as to categorize their epilepsy syndrome according to the 1989 ILAE classification [13]. This classification system utilizes two axes (localization and etiology) in order to categorize epilepsy syndromes. With regard to localization, epilepsies are classified as focal (synonymous terms: partial- or localization-related) and generalized. Regarding etiology, epilepsies are classified as idiopathic, symptomatic or cryptogenic. The latter are defined as epileptic syndromes that are believed to be symptomatic, but no etiology can currently be identified.

Study inclusion criteria were as follows: (1) No clinical seizure for at least 7 days prior to study entry. As most cases of ictal and post-ictal anxiety and depression abate within 2–3 days [14], the focus of the present study was exclusively on interictal mood disorders. It should be noted that the distinction between interictal (i.e. occurring in the periods inbetween epileptic seizures) and ictal and post-ictal mood disorders is a crucial one, because they differ regarding the underlying pathophysiological mechanisms. The former might represent psychological worries about the occurrence of seizures and their possible consequences whereas the latter are directly related to epileptic discharges. (2) No history of status epilepticus for at least 6 months prior to study entry. (3) No history of psychotropic medication intake including benzodiazepines. (4) No history of substance or alcohol abuse. (5) A Mini-Mental State Examination (MMSE) > 24. Thereby, all patients with significant cognitive dysfunction were excluded from the study. The treating neurologists made every possible effort to ensure that patients did not experience any CNS side effects from their antiepileptic medication at the time of psychological testing that might interfere with the assessment.

All subjects were administered the following instruments: (1) Beck Depression Inventory, 21 item version (BDI-21), a widely used and well validated 21-item self-report inventory of depressive symptoms [15]. The BDI-21 score ranges from 0 to 63. (2) State-Trait Anxiety Inventory (STAI), an extensively used self-administered inventory of two sections containing 20 items each, designed to explore anxiety in its state and trait dimensions [16]. The minimum score for each section is 20, with a maximum score of 80. (3) MMSE [17]. As previously noted, all patients with a MMSE score < 24 were excluded from further evaluation.

### Statistical analysis

Continuous data are presented as mean ± standard deviation (SD) while non-continuous variables are given as percentages.

In order to assess which factors are independently associated with BDI-21, STAI-S and STAI-T, a two-step approach was adopted. As a first step, simple regression analyses were performed with various factors such as age, gender, the type of epilepsy syndrome, number of antiepileptic drugs and disease duration selected as independent variables while BDI-21 and STAI-S and -T were selected as dependent variables. Subsequently, the most significant of these factors were further investigated with multiple regression analysis. Data concerning age, duration of disease, BDI-21 and STAI were entered in the model as continuous variables, while the variables of gender, epilepsy type and medication were entered in the model as dummies variables. Seizure frequency was abbreviated as SF and the type of epileptic syndrome was denoted as CFE for cryptogenic focal epilepsy, SFE for symptomatic focal epilepsy and IGE for idiopathic generalized epilepsy, Medication was coded as AED1–3 indicating the use of 1–3 antiepileptic drugs, respectively.

The associations between dependent and independent variables are presented by means of unstandardized linear regression coefficients and 95% confidence intervals. In addition, all reported associations were ranked according to the absolute value of their standardized effect, which was quantified by the standardized regression coefficients (β). A standardized regression coefficient is defined as a regression coefficient that has the effect of the measurement scale removed so that the size of the coefficient can be interpreted; it is calculated by multiplying the regression coefficient by the ratio of the standard deviation (SDx) of the independent variable to the standard deviation (SDy) of the dependent variable (β = regression coefficient × SDx/SDy).

For all tests, p < 0.05 was the level of significance. Statistical analysis was performed using a commercially available statistical package (SPSS for Windows version 13; SPSS, Chicago, IL, USA).

## Results

Table 1 presents the demographic data and principal characteristics of our sample (n = 201). The age of the patients ranged from 16–60 years with a mean value of 33.2 years. The BDI-21 score had a mean value of 7.6 ± 7.3 with 32% of the study population having scores ≥ of 15, which is the cut-off point of the Greek version of the BDI-21 [18]. STAI-S and STAI-T had mean scores of 48.6 ± 6.7 and 42.9 ± 6.7, respectively, and both were significantly increased compared to control values obtained in healthy volunteers (24.95 ± 11.36 for the state and 27.88 ± 11.43 for the trait score, p < 0.001).

**Table 1:** Demographic data and principal characteristics of the study sample (n = 201)

**Variable**	**Mean ± SD/%**	**Variable**	**Mean ± SD/%**
STAI-S	48.6 ± 6.7	Seizure frequency > 1/year	44.3%
STAI-T	42.9 ± 6.7	Cryptogenic focal epilepsy	27.4%
BDI-21	7.6 ± 7.3	Symptomatic focal epilepsy	51.2%
Age	33.2 ± 10.0	Idiopathic generalized epilepsy	21.4%
Disease duration	13.9 ± 9.5	Antiepileptic drug 1	50.2%
Male gender	51.2%	Antiepileptic drug 2	34.8%
Seizure frequency > 1/month	23.9%	Antiepileptic drug 3	14.9%
Seizure frequency < 1/year	31.8%		

Non-continues variables are presented as percentages. Continuous variables are presented as Mean ± SD.

**Figure 1:**
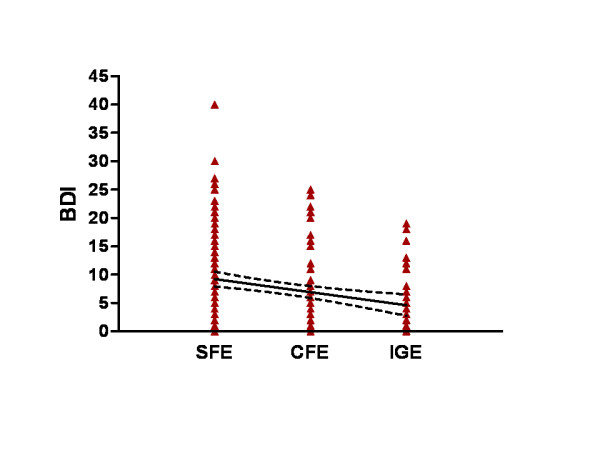
**Scattergram demonstrating the relationship between BDI score and the type of epilepsy syndrome**. SFE: symptomatic focal epilepsy, CFE: cryptogenic focal epilepsy, IGE: idiopathic generalized epilepsy. A linear regression line and the 95% confidence band are shown. Slope: -2.299 ± 0.6290 (95% confidence interval: -3.532 to -1.066); y-intercept: 11.50 ± 1.182 (95% CI: 9.187 to 13.82); p < 0.001.

Table 2 presents the estimated association levels of depression quantified using the BDI-21 index, with demographic and clinical characteristics that were evaluated using simple linear regression analyses. Variables such as seizure frequency, type of epilepsy syndrome and number of antiepileptic drugs were significantly (p < 0.01) associated with BDI-21. More specifically, BDI-21 was positively associated with symptomatic focal epilepsy (β = 3.60, p < 0.001) and negatively with idiopathic generalized epilepsy (β = -3.35, p = 0.007) (Figure 1). High seizure frequency (SF > 1/month) and a high number of antiepileptic drugs (AED3) were positively associated with depression (β = 4.88, p < 0.001 and β = 3.065, p = 0.034, respectively) whereas a low number of antiepileptic drugs (AED1) showed a negative association with the depression index (β = -2.52, p = 0.014). Finally, female gender showed a trend towards being significantly associated with the BDI-21 index (β = 1.99, p = 0.054).

**Table 2:** Simple linear regression analyses of factors associated with BDI-21

		**95% Confidence interval**				
					
**Factor**	**Unstandardized coefficient**	**Lower bound**	**Upper bound**	**Standardized coefficients**	**p Value**	**R^2^**
Gender	1.991	-0.036	4.018	0.136	0.054	0.018
Seizure frequency > 1/month	4.888	2.588	7.187	0.285	< 0.001	0.081
Seizure frequency < 1/year	-2.129	-4.305	0.046	-0.136	0.055	0.018
Seizure frequency > 1/year	-1.728	-3.773	0.317	-0.117	0.097	0.014
Symptomatic focal epilepsy	3.605	1.621	5.588	0.246	< 0.001	0.061
Cryptogenic Focal Epilepsy	-1.691	-3.973	0.591	-0.103	0.145	0.103
Idiopathic generalized epilepsy	-3.357	-5.806	-0.907	-0.188	0.007	0.035
Antiepileptic drug 1	-2.523	-4.538	-0.508	-0.172	0.014	0.030
Antiepileptic drug 1	1.064	-1.077	3.206	0.069	0.328	0.005
Antiepileptic drug 1	3.065	0.227	5.904	0.149	0.034	0.022
Age	-0.040	-0.142	0.062	-0.054	0.443	0.003
Duration of disease	-0.036	-0.145	0.073	-0.046	0.516	0.002

To determine whether the association between seizure frequency and BDI-21 index was independent of the type of epilepsy syndrome (SFE), a backward multiple regression analysis was performed. The results of the multiple regression analysis are presented in Table 3 and reveal that the unstandardized coefficients of both SF > 1/month (β = 4.35, p = 0.001) and SFE (β = 3.05, p = 0.002) were independent determinants of BDI-21. The predicted multiple regression model accounted jointly for 12.4% of the variation of the BDI-21 (R^2 ^= 0.124).

**Table 3:** Multiple linear regression of factors associated with BDI-21

		**95% Confidence interval**				
					
**Factor**	**Unstandardized coefficient**	**Lower bound**	**Upper bound**	**Standardized coefficients**	**p Value**	**Tolerance**
(Constant)	4.990	3.559	6.420		0.001	
Seizure frequency > 1/month	4.353	2.076	6.630	0.254	0.001	0.978
Symptomatic focal epilepsy	3.050	1.107	4.402	0.208	0.002	0.978

The base omitted factors are gender, SF < 1/year, SF > 1/year, CFE, IGE, AED1, AED2, AED 3, age and disease duration.

The associations of levels of anxiety quantified using STAI-S with demographic and clinical characteristics is presented in Table 4. It is concluded that only the type of epilepsy is significantly associated with the STAI-S index (p < 0.001). In particular, SFE was positively correlated to STAI-S (β = 4.39, p < 0.001), whereas CFE showed a negative correlation (β = -3.675, p < 0.001). The results of multiple regression analysis, however, showed that only SFE was an independent determinant of STAI-S (β = 4.391, 95% CI 2.625–6.156, p < 0.001). The predicted multiple regression model accounted for 10.4% of the variation of the STAI-S (R^2 ^= 0.108).

**Table 4:** Simple linear regression analyses of factors associated with STAI-S

		**95% Confidence interval**				
					
**Factor**	**Unstandardized coefficient**	**Lower Bound**	**Upper Bound**	**Standardized coefficients**	**p Value**	**R^2^**
Gender	1.603	-0.253	3.458	0.120	0.090	0.009
Seizure frequency > 1/month	1.186	-0.999	3.371	0.076	0.286	0.006
Seizure frequency < 1/year	1.073	-0.927	3.073	0.075	0.291	0.006
Seizure frequency > 1/year	-1.818	-3.681	0.046	-0.135	0.056	0.018
Symptomatic focal epilepsy	4.391	2.625	6.156	0.328	< 0.001	0.108
Cryptogenic focal epilepsy	-3.675	-5.707	-1.644	-0.245	< 0.001	0.060
Idiopathic generalized epilepsy	-2.180	-4.437	0.078	-0.134	0.058	0.018
Antiepileptic drug 1	-1.618	-3.473	0.237	-0.121	0.087	0.015
Antiepileptic drug 2	1.122	-0.832	3.077	0.080	0.259	0.006
Antiepileptic drug 3	1.179	-1.438	3.796	0.063	0.375	0.004
Age	0.001	-0.093	0.094	0.001	0.988	0
Duration of disease	0.096	-0.002	0.194	0.136	0.055	0.019

**Figure 2:**
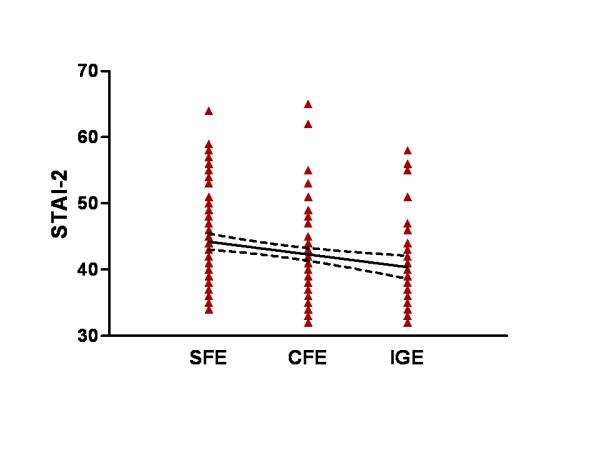
**Scattergram demonstrating the relationship between STAI-2 score and the type of epilepsy syndrome**. SFE: symptomatic focal epilepsy, CFE: cryptogenic focal epilepsy, IGE: idiopathic generalized epilepsy. A linear regression line and the 95% confidence band are shown. Slope: -1.932 ± 0.5797 (95% confidence interval: -3.068 to -0.7957); y-intercept: 46.16 ± 1.090 (95% CI: 44.03 to 48.30); p < 0.001.

The associations of levels of anxiety quantified using STAI-T index with demographic and clinical characteristics are presented in Table 5. Variables such as seizure frequency > 1/month and > 1/year, as well as SFE, CFE and the patient's gender were significantly associated with STAI-T index (p < 0.001). In particular, high seizure frequency (SF > 1/month (β = 3.85, p < 0.001)), polypharmacy (AED3 (β = 3.242, p = 0.015), SFE (β = 3.52, p < 0.001) (Figure 2) and female gender (β = 2.85, p = 0.002) were positively correlated to the STAI-T. In contrast, low seizure frequency (SF > 1/year, β = -2.761, p = 0.004), use of monotherapy (AED1, β = -2.277, p = 0.016) and cryptogenic focal epilepsy (CFE, β = -2.657, p = 0.012) were found to be negatively correlated with the STAI-T index.

**Table 5:** Simple linear regression analyses of factors associated with STAI-T

		**95% Confidence interval**				
					
**Factor**	**Unstandardized coefficient**	**Lower bound**	**Upper bound**	**Standardized coefficients**	**p Value**	**R^2^**
Gender	2.851	1.018	4.684	0.212	0.002	0.045
Seizure frequency > 1/month	3.858	1.726	5.990	0.245	< 0.001	0.060
Seizure frequency < 1/year	-0.093	-2.105	1.920	-0.006	0.928	0
Seizure frequency > 1/year	-2.761	-4.609	-0.913	-0.204	0.004	0.042
Symptomatic focal epilepsy	3.521	1.711	5.331	0.262	< 0.001	0.069
Cryptogenic focal epilepsy	-2.657	-4.727	-0.587	-0.177	0.012	0.031
Idiopathic generalized epilepsy	-2.090	-4.358	0.178	-0.128	0.071	0.016
Antiepileptic drug 1	-2.277	-4.126	-0.429	-0.170	0.016	0.029
Antiepileptic drug 2	0.695	-1.271	2.661	0.049	0.487	0.002
Antiepileptic drug 3	3.242	0.649	5.834	0.172	0.015	0.030
Age	-0.032	-0.126	0.062	-0.048	0.499	0.002
Duration of disease	0.047	-0.053	0.147	0.066	0.356	0.004

To determine whether the association between seizure frequency and STAI-T, as well as between gender and STAI-T were independent of the type of epilepsy, multiple regression analysis was employed. The estimated coefficients of the multiple regression analysis are presented in Table 6 and reveal that only high seizure frequency (β = 3.56, p = 0.001), SFE (β = 2.61, p = 0.004) and female gender (β = 2.56, p = 0.005) were independent determinants of STAI-T. The predicted multiple regression model account jointly for 14.7% of the variation of the STAI-T (R^2 ^= 0.147).

**Table 6:** Multiple linear regression of factors associated with STAI-T

		**95% Confidence interval**				
					
**Factor**	**Unstandardized coefficient**	**Lower bound**	**Upper bound**	**Standardized coefficients**	**p-Value**	**Tolerance**
(Constant)	36.868	34.041	39.694		0.001	
Seizure frequency > 1/month	3.568	1.496	5.640	0.227	0.001	0.971
Symptomatic focal epilepsy	2.617	0.825	4.409	0.195	0.004	0.944
Gender	2.564	0.790	4.339	0.191	0.005	0.963

The base omitted factors are SF < 1/year, SF > 1/year, CFE, IGE, AED1, AED2, AED 3, age and disease duration.

## Discussion

The present cross-sectional study investigated the association of certain demographic and seizure-related variables with mood disorders in patients with epilepsy. Our results confirm the prevailing view that depression and anxiety are common psychological disorders in epileptics. It is additionally concluded that female gender, high seizure frequency and a symptomatic epilepsy syndrome are independent, positively associated factors for the development of anxiety and/or depression.

The effect of gender on the development of psychiatric disturbances in epilepsy has been highly controversial in previous studies, most likely due to diverse methodological approaches and differences in the investigated populations. With regard to depression, Altshuler et al [7] observed that male patients with temporal lobe epilepsy had the highest scores on the BDI-21, and Strauss et al [6] observed that men with left-sided temporal foci were more vulnerable to depression. Other investigators have also concluded that men are overrepresented in depressed patients [12,19,20] and in groups of patients with self-destructive tendencies [21]. By contrast, a number of studies reported opposite results with gender having either no effect [22,23] or with a preponderance (though not statistically significant) of female patients with epilepsy and depression [8]. Our results are in line with this latter study, showing a trend towards significant association between female gender and depression in epilepsy. The lack of a clear-cut gender difference in the prevalence of depression among epilepsy patients is worth noting in view of the fact that female gender is a recognized risk factor for depression in non-epileptic populations [1].

With regard to anxiety in epilepsy, gender is generally considered to have a subtle effect [24] with the notable exception of the study by Jacoby et al [2], which concluded that female patients tend to be more anxious than men. Our results reveal an interesting novel finding, namely that the effect of gender critically depends on the specific aspects of anxiety investigated. In the present study we administered STAI [16], which is a self-completed questionnaire consisting of two different 20-item forms. The former (STAI-S) measures various subjective and somatic manifestations of anxiety at a given moment. In contrast, the latter (STAI-T) refers to relatively stable individual differences in anxiety proneness as a personality trait [25]. Our results indicate that female patients had significantly higher scores on STAI-T compared to males. This finding is reminiscent of the pattern occurring in normal subjects as trait scores have been previously reported to be more common in women [26]. Therefore, it most likely reflects a tendency observed in the general population. In contrast, no gender difference was disclosed regarding STAI-S, and therefore interictal anxiety in its state form is not related to gender. Previous studies have attributed interictal anxiety to a combination of biological factors (i.e. seizure-induced alterations of neuronal circuits in the amygdala region via a kindling-like mechanism) [27] and psychological worries concerning, for instance, the possibility of seizure-related injuries or the impact of epilepsy on employment and marital status [28,29].

Seizure frequency has been linked to psychological disturbances in a number of relevant studies. With regard to depression, Boylan et al [30] reported that 50% of inpatients undergoing video-EEG telemetry suffered from depression with 19% exhibiting suicidal ideation. Jacoby et al [2] observed in a community-based survey that 21% of patients with recurrent seizures were depressed versus 9% of controlled subjects and O'Donoghue et al [31] have similarly demonstrated at primary care level that 33% of patients with recurrent seizures versus 6% of those in remission had probable depression. Overall, the prevalence of depression has been reported to range from 20 to 55% in pharmacoresistant populations versus 3–9% in well controlled subjects [32]. Parenthetically, the occurrence of depression in epileptic patients, particularly those with high seizure counts, might seem paradoxical as one of the most powerful treatments for depression is electroconvulsive therapy, which is entirely based on the tenet of the antidepressive effects of convulsions.

Regarding anxiety, Smith et al [33] classified 33% of drug-resistant epileptics recruited from a referral center as clinically anxious with a group mean score of 7.7 in the Hamilton Anxiety and Depression scale. In contrast, Jacoby et al [2] reported that 25% of patients in a large community-based study suffered from anxiety with a group mean of 6.8 on the HAD scale and ascribed the lower prevalence figures of anxiety in their study to the fact a large percentage of patients were either seizure-free or experiencing infrequent seizures. Our results are in line with the above-mentioned views indicating a positive association between high seizure frequency and increased scoring on the BDI-21, STAI-S and STAI-T scales. It should be noted that this association is not a direct one as ictal and post-ictal anxiety and depression have been *a priori *excluded by the design of the present study. It rather indicates that as the burden of epilepsy increases, so does the severity of mood disorders.

The important issue of the relationship between specific epilepsy syndromes and the development of mood disorders has not been thoroughly addressed in the past, as most of the relevant studies have analyzed seizure subtypes and etiology as separate factors while very few have utilized a syndromic approach. Our data suggest that cryptogenic or symptomatic focal epilepsies are positively associated with the presence of psychological disturbances. This finding was anticipated, to some degree, in view of the results of previous studies. Partial seizures are a hallmark of focal epilepsies, and a number of investigations have clearly established that partial seizures, particularly complex partial seizures of temporal lobe origin, are a risk factor for the development of depression and anxiety [6,12,34-37].

The effect of seizure etiology, however, has been rather inconsistent. For instance, some [9,10], but not all [11,12], relevant studies have reported that depression is more common in epileptic patients with a structural lesion. Our finding that symptomatic epilepsies are associated with increased anxiety and depression is certainly in line with the former ones. This view is intuitively correct, as depression is commonly associated with neurological conditions (i.e. stroke or head injury) that are also responsible for epilepsy.

In conclusion, our results confirm the generally held view that mood disorders are common in patients with newly diagnosed and chronic epilepsy and provide further insight to the association of depression and anxiety with certain demographic and seizure-related variables.

## Competing interests

The author(s) declare that they have no competing interests.
